# Supplementary material for: Dosimetry of a Novel 111Indium-Labeled Anti-P-Cadherin Monoclonal Antibody (FF-21101) in Non-Human Primates
Source: Cancers (Basel). 2023 Sep 13;15(18):4532. doi: 10.3390/cancers15184532 (PMC10526467; doi:10.3390/cancers15184532)
Supplement: Supplementary file 1 [file cancers-15-04532-s001.zip › cancers-2527598-supplementary.pdf]

The following dosimetric assumptions were made regarding FF-21101(^{111}In) and FF-21101(^{90}Y)

1. Once injected, the radiolabeled antibodies remained intact.
2. Defined NHP source organs of activity were lungs, heart contents, liver, spleen, kidneys, red marrow and testes (in males), and the remainder of the body (retained total body activity minus that within the defined sources, assumed to be uniformly distributed throughout the remaining body tissues.)
3. All organ activity versus time exhibited single exponential clearance, with assumed instantaneous uptake:

$$f_s(t) = f_s(0) \times e^{-\ln(2)\, t / T_{Seff}}$$
$$T_s = f_s(0) \times T_{Seff} / \ln(2)$$

4. Red marrow activity was derived from fraction of injected activity (FIA) FIA/ml in whole blood, using the well-established Sgouros formula for radiolabeled antibodies [39]:

$$f_{sMarrow}(t) = \frac{C_{blood}(t) \times 019 \times M_{marrow}}{(1 - HCT)}$$

where $C_{blood}(t)$ = scaled NHP FIA/ml of whole blood at time t (i.e., NHP FIA/ml times the NHP-to- reference adult male or female total body mass ratio), M_{marrow} = reference adult mass of red marrow from OLINDA/EXM 1.1 (male: 1120 g, female: 1300 g), and HCT = average normal human hematocrit (male: 0.47, female: 0.42).

5. The estimated human blood FIA/mL was further conservatively scaled so that the total blood fraction of activity was 100% of the administered activity at time t = 0.
6. Source organ residence time was converted from that for the NHP to that for the corresponding reference adult human (male or female) using an established formula for scaling by relative organ–to-total body mass ratio [40]

$$\frac{NHP_{total}}{NHP_{organ}} \times \frac{Human_{organ}}{Human_{total}}$$

where individual NHP measured total body mass and source organ masses estimated from the defined VOIs multiplied by an assumed organ tissue density (lung: 0.25 g/ml; other source organs: 1.03 g/ml), were employed; and the reference adult male and female total body and organ masses are those from OLINDA/EXM 1.1 (except whole blood masses, which were obtained from ICRP 89) [41].

7. FF-21101(^{90}Y) residences times were then derived from those for FF-21101(^{111}In), assuming identical biodistribution and using the formula for T_{Seff}. That is:

$$\frac{1}{T_{1/2bio}} = \frac{1}{T_{Seff}} - \frac{1}{T_{1/2phys\,^{111}In}}$$

$$\frac{1}{T_{S_{eff\,^{90}Y}}} = \frac{1}{T_{1/2bio}} + \frac{1}{T_{1/2phys\,^{90}Y}}$$

$$T_s = f_s(0) \times T_{Seff\,^{90}Y} / \ln(2)$$

8. Human radiation absorbed dose estimates per unit of administered activity, for both FF-21101(^{111}In) and FF-21101(^{90}Y), were calculated for each individual NHP by the OLINDA/EXM 1.1 program, based on the corresponding radionuclide, derived source organ and remainder of body (total body minus all source organ) residence times and NHP gender.

The calculated absorbed doses of FF-21101(^{111}In) and FF-21101(^{90}Y) were then compared with the published absorbed doses for Zevalin labeled with the same radionuclide (^{111}In)- and (^{90}Y)- ibritumomab tiuxetan, Spectrum Pharmaceuticals Inc., Irvine, CA).

References:

39. Sgouros, G. Bone marrow dosimetry for radioimmunotherapy: theoretical considerations. J. Nucl Med. 1993, 34, 689-694.
40. Macey, D.J.; Williams, L.E. AAPM Report No 71, A Primer for Radioimmunotherapy and Radionuclide Therapy; Medical Physics Publishing: Madison, WI, USA, 2001
41. ICoR Protection. Basic Anatomical and Physiological Data for Use in Radiological Protection: Reference Values; ICRP Report No. 89; ICRP Publication: Pergamon, Turkey; Oxford, UK; New York, NY, USA, 2001.
